# The impact of feeding pattern on heavy metal accumulation and associated health risks in fishes from the Dhaleshwari River Bangladesh

**DOI:** 10.1016/j.heliyon.2024.e40509

**Published:** 2024-11-19

**Authors:** Evena Parvin Lipy, Liton Chandra Mohanta, Dipa Islam, Chadni Lyzu, Samina Akhter, Mahmuda Hakim

**Affiliations:** Biomedical and Toxicological Research Institute (BTRI), Bangladesh Council of Scientific and Industrial Research (BCSIR), Dhanmondi, Dhaka, 1205, Bangladesh

**Keywords:** Chromium pollution, Dhaleshwari river, Carnivorous fish, Noncarcinogenic and carcinogenic risk assessment, Seasonal variation

## Abstract

Heavy metals in aquatic ecosystems accumulate in fish tissues, posing significant ecological and health hazards due to their toxic effects on both the environment and human consumers. The purpose of the study was to assess the potential hazards associated with consuming carnivorous, omnivorous, and herbivorous fish species from the Dhaleshwari River in Bangladesh. The study focused on the seasonal variation and accumulation pattern of toxic heavy metals in these fish species. For this, four fish species (*Wallagu attu*, *Ompak bimaculatus, Labeo calbasu, Cirrhinus mrigala* were sampled during the dry and wet seasons from the contiguity of the industrial outlet from Dhaleshwari River. The amount of accumulation of Cr, Pb, Cd, Cu and As in the muscles of the samples were analyzed using spectrophotometry. Heavy metal concentrations were observed to be in the following descending order: Cu (1.99) > Cr (1.92) > Pb (1.42) > Cd (0.31) > As (≤0.0002) (mg/kg, wet weight). Cr, Cd and Pb concentrations exceeded some international food safety guidelines for fish muscles. Carnivorous species exhibited higher metal accumulation than omnivores and herbivores. Statistical analysis revealed significant variations among seasons (p < 0.001), species (p < 0.05) and strong correlation among metals (except As) with p < 0.01. Risk assessment suggested carnivorous species might pose noncarcinogenic risks to both of its child and adult consumers. Despite the potential noncarcinogenic risks, the alarming levels of chromium in these fish indicate a substantial cancer risk for both adults and children, raising concerns about the safety of consuming fish from the Dhaleshwari River. These findings strongly emphasize the importance of implementing enhanced regulatory monitoring to mitigate health risks associated with contaminated fish consumption.

## Highlights

• Carnivorous species tend to accumulate more heavy metals than others.
• Seasonal and species variation was observed.
• Cr, Pb and Cd concentrations in fish muscles exceeded international safety limits.
• High Cr concentration pose serious risk for fish consumers in long term exposure.
• Children are at high risk than adults.

## Graphical abstract

**Figure:**
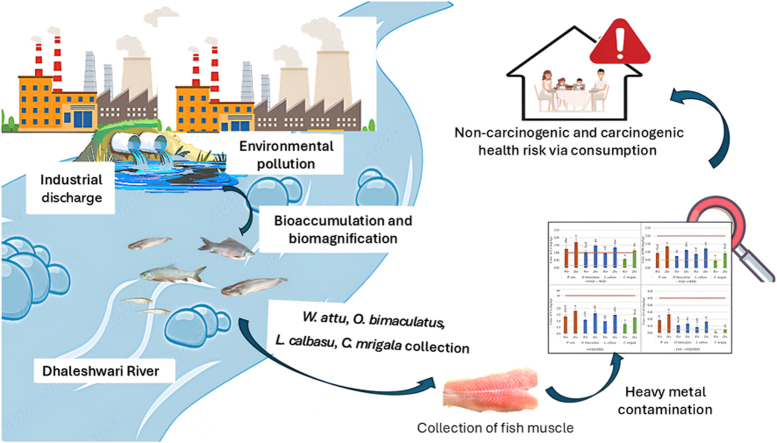

## 1 Introduction

Fish is a popular well-balanced diet all over the world because it acts as an essential source of proteins, vitamins, minerals, unsaturated fatty acids, energy, etc. [1,2]. Global fish production in 2018 estimated to be 179 million tons, with 156 million tons allocated for human consumption and the remaining 22 million tons entirely used for fish meal and fish oil manufacturing [3]. Conversely, humans may be susceptible to heavy metals (HMs) through the food chain by consuming fish. Fish are capable of accumulating and biomagnifying HM in their bodies as a result of their position at the highest point of the food chain [2]. The occurrence of HM contamination in aquatic habitats has emerged as a significant health issue as it has the probability to be transported into the digestive tracts through ingestion and finally affect various organ systems [4]. For instance, Cr can cause alteration of genetic materials, degeneration of immune systems, neurotoxicity, nephrotoxicity, skin and lung cancer [5]. Lead poisoning can impede the cognitive development of children and result in acute and chronic impairments to the renal, reproductive, cerebral, and nervous systems [6,7]. Cadmium can cause harm to human health, affecting multiple organs and systems, including the lungs, liver, placenta, endocrine tissues, kidneys, bones, heart, and reproduction [8]. Inorganic arsenic may cause problems to the skin, liver, respiratory and gastrointestinal tract, cardiovascular system and nervous system as well [9,10].

The capital of Bangladesh, Dhaka, is encompassed by several rivers and canals. The Dhaleshwari River, a 160-km tributary of the Jamuna River, has long served as a crucial source of livelihood for the local population in Bangladesh [11,12]. Its fertile waters and abundant fish stocks have supported both subsistence fishing and commercial fisheries, contributing significantly to the region's economy. Its strategic location has also contributed to the region's economic development. However, the establishment of industries in the Dhaka Export Processing Zone (EPZ) and the relocation of tanneries from Hazaribagh have led to significant pollution of the river [13]. These industries release substantial quantities of solid waste and effluents into the Dhaleshwari River, including 232 tons of solid waste and 20,000 cubic meters of tannery effluents daily [14]. Improper waste management practices, such as the dumping of solid waste near the tannery estate and the partial operation of the central Effluent Treatment Plant (ETP), have further exacerbated the river's pollution [15]. Tanning processes involve the use of harmful chemicals, including chrome salts, which contribute to heavy metal pollution in the river and bioaccumulation of Cr in fish of the adjacent aquatic environment [16]. Previous studies [13] have identified elevated levels of Cr, Cd, and Pb in the Dhaleshwari River, surpassing international safety limits. These contaminants have been found to bioaccumulate in commonly consumed fish species, posing a significant health risk to consumers. Additionally, the concentrations of these metals have been observed to vary among different fish species and across seasons. To gain a deeper understanding of the factors influencing metal accumulation, such as the feeding patterns of fish species, seasonal variations, and the associated risks in various age groups, further research is necessary.

The aim of this study was to assess the concentrations of Cr, Pb, Cd, Cu and As in the muscles of carnivorous (*W. attu*), omnivorous (*O. bimaculatus and L. calbasu*), and herbivorous *(C. mrigala)* fish species collected from the Dhaleshwari River. The study also conducted a comparison between the accumulated metal concentrations in these fish and internationally accepted safety limits. Additionally, it examined the impact of feeding habits on the bioaccumulation of these metals during wet and dry seasons. Furthermore, the study evaluated the associated carcinogenic and non-carcinogenic hazards for both adults and children who consume these fish species.

## 2 Materials and methods

### 2.1 Sampling

**Fig. 1:**
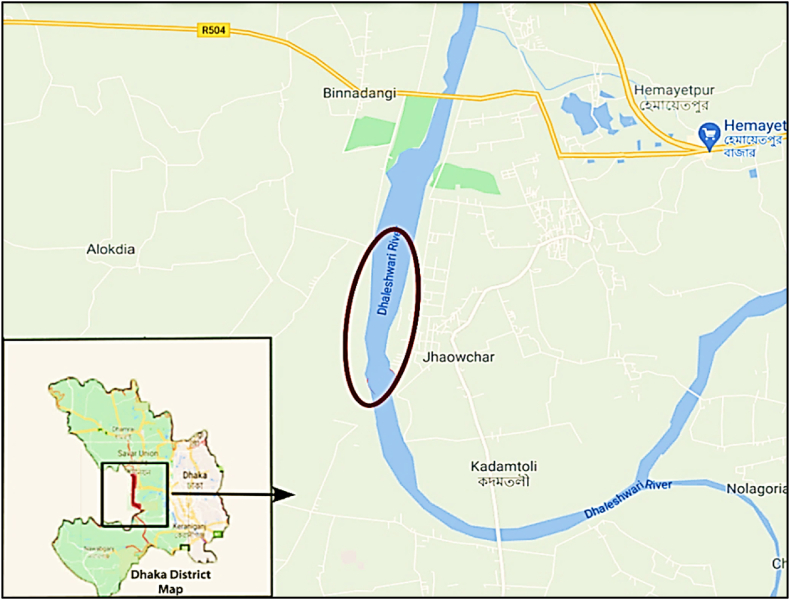
The studied area of Dhaleshwari River, Bangladesh.

To focus on the tannery pollution, the study area was selected closer to the central Effluent Treatment Plant (ETP) of Dhaka EPZ, specifically 23°47′05.2″N 90°14′23.9″E to 23°46′02.4″N 90°14′15.2″E (Fig. 1). Four commonly consumed fish species having different feeding habits, namely *Wallagu attu* (Boal), *Ompak bimaculatus* (Pabda), *Labeo calbasu* (Kalibaus) and *Cirrhinus mrigala* (Mrigel) were collected in triplicate from professional fishermen at the sampling area in each season. To assess the seasonal variation among them, these fish species were sampled during dry and wet seasons. Following collection, samples were transported in an icebox to the laboratory for heavy metal analysis. After cleaning with tap water, the muscles were dissected with a sharp scalpel. These samples were labeled carefully and stored in a −20 °C freezer for subsequent analysis.

### 2.2 Heavy metal extraction and analysis

Before analysis, frozen fish muscles were thawed and freeze dried. Samples were ground with mortar and pestle to get a homogenous mixture. Following a previously described method [13], each dried sample (0.3 g) was placed into a Teflon tube together with 69 % HNO_3_ acid (5 mL) and 30 % H_2_O_2_ (2 mL). It was subsequently digested in an automated microwave digestion system (Model: Milestone-ETHOS1). After digestion, samples were then filtered and mixed with ultrapure water to the desired volume. Extracted samples were analyzed using Flame Atomic Absorption Spectrophotometer (Model: Shimadzu AA-7000) to determine the contamination of Cr, Pb, Cd and Cu. However, Graphite Furnace Atomic Absorption Spectrophotometer (Model: Shimadzu GFA-7000) was used to determine the As contamination.

### 2.3 Quality assurance

Samples were carefully weighed using a weighing scale (Model: Mettler Toledo ML204). Standards and solutions were prepared with ultrapure water and chemicals used were analytical grade procured from Merck, Germany. Before use, each glassware was soaked in 20 % HNO_3_ overnight and rinsed with Ultrapure water. Quality was assured by considering distinct factors, i.e., blanks, calibration curve, and recovery analysis [17]. All calibration curves exhibited a high correlation coefficient (R^2^) of over 0.998. The limit of quantification (LOQ) for analyzing Cr, Pb, Cu, Cd and As were found to be 0.11, 0.10, 0.0850, 0.2, and 0.0040 mg/kg, respectively.

### 2.4 Risk assessment

#### 2.4.1 Estimated daily intake (EDI)

Each metal's daily intake through fish consumption was assessed using the following equation:

(1) $$\text{EDI}=\frac{EFr\;X\;ED\;X\;FIR\;X\;Cm}{BW\;X\;TA}\;X\;10^{-3}$$

Where, EFr, ED and TA denote the exposure frequency (365 days per year), duration of exposure equivalent to the life span of an average Bangladeshi person (72.3 years) and the average exposure time (365x72.3) respectively. *Cm* represents the mean concentration (mg/kg, ww) of metals in fish, FIR refers to the daily fish consumption rate by an average Bangladeshi (67.58 g/day) [18], and BW refers to the mean body weight of a Bangladeshi (adult: 60 kg and child: 15 kg) [19,20].

#### 2.4.2 Noncarcinogenic risk

To evaluate the noncarcinogenic risk for an individual through the ingestion of fish, the Target Hazard Quotient (THQ) was calculated. It refers to the threshold (RfD) of a contaminant exposure through a single exposure pathway below which an individual has a tendency to experience the noncarcinogenic effects on health. It is to be mentioned that it was assumed that all consumed HMs were completely absorbed by the person; cooking did not alter the contaminant in the fish [21,22]. Therefore, THQ values were measured by the following equation [23,24].

(2) $$\text{THQ}=\frac{EFr\;X\;ED\;X\;FIR\;X\;Cm}{RfD\;X\;BW\;X\;TA}\;X10^{-3}=\frac{EDI}{Rfd}$$

Here, FIR, *Cm*, EFr, ED, TA and BW denote the same factors as mentioned above. RfD denotes the reference dose (mg/kg day) via oral route for different HMs [RfD value for Cr (0.003), Pb (0.004), Cu (0.04), Cd (0.001) and As (0.0003) in mg/kg day] [25,26].

#### 2.4.3 Hazard index (HI)

Exposure of a human to several toxicants results in a cumulative effect known as the Hazard index, which can be analytically determined by summing up the individual THQ values [26]. Therefore, for this study HI was calculated as follows:

HI = THQ_Cr_ + THQ_Pb_ + THQ_Cd_ + THQ_Cu_ + THQ_As_

Where, THQ_Cr_, THQ_Pb_, THQ_Cd_, THQ_Cu_ and THQ_As_ denote the individual THQ of respective HMs.

#### 2.4.4 Target carcinogenic risk (TR) assessment

The target carcinogenic hazards in the fish samples obtained by consuming heavy metals were determined using the following numerical formula [25,27].

$$\text{TR}=\frac{EFr\;X\;ED\;X\;FIR\;X\;C\;X\;CSF}{BW\;X\;TA}\;X10^{-3}$$

Here, EFr, ED, FIR, C, BW and TA hold the same expression mentioned in the previous section. The cancer slope factor, or CSF, is a tool that may be used to assess risk probability for various exposure levels. It provides an upper-bound assessment of risk per increase in dose [28]. Cr, Pb, Cd and As have the CSF values of 0.5, 0.0085, 0.38, and 1.5 mg/kg day [21,27].

### 2.5 Statistical analysis

The statistical analyses were carried out using IBM SPSS Version 22 application. Analysis of the data included descriptive statistics, t-testing, the analysis of variance (Levene's test for significance (p ≤ 0.05), Tukey's HSD method (p ≤ 0.05), and Pearson's correlation coefficient (p ≤ 0.05, 0.01).

## 3 Results and discussion

### 3.1 Ecological characteristics and moisture content in fish species

The ecological characteristics and moisture content of the collected fish species, namely, *Wallagu Attu, Cirrhinus mrigala*, *Ompok bimaculatus* and *Labeo calbasu* were listed in Table 1. Their characteristics revealed that, *W. attu* is carnivorous, whereas *C. mrigala is* herbivorous and *O. bimaculatus* and *L. calbasu* are omnivorous in nature. Two omnivore species were taken because of their mixed feeding patterns.

**Table 1:** Ecological characteristics and moisture content of the fish species.

Scientific name	Local name	Feeding habit	Biotype complex	Moisture content	References
*Wallagu attu*	Boal	Carnivore	Bottom dwellers	79.00 %	[29]
*Ompak bimaculatus*	Pabda	Omnivore	Upper level of water	75.58 %	[30]
*Labeo calbasu*	Kalibaus/Kalbasu	Omnivore	Bottom dwellers; occasionally comes to surface	77.27 %	[31]
*Cirrhinus mrigala*	Mrigel	Herbivore	benthopelagic/bottom dwellers	74.92 %	[32]

### 3.2 Accumulation of HMs in fish muscles

**Fig. 2:**
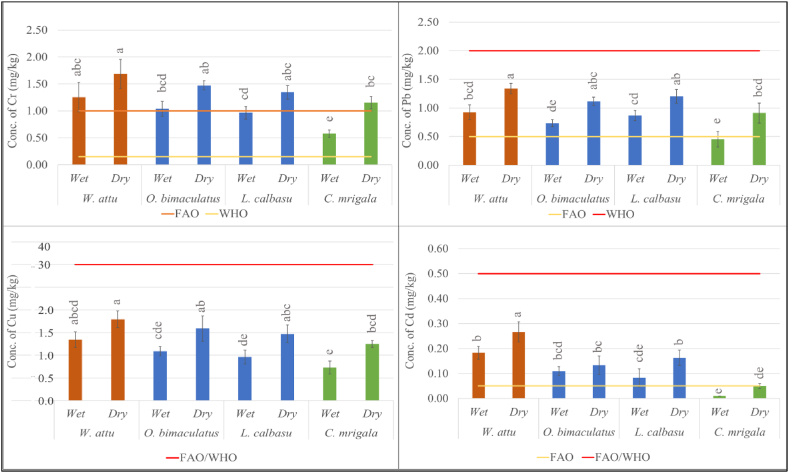
Concentrations of HMs in sampled fish species in two seasons and comparison of the resultant concentrations with international safety standards. A distinct alphabetic letter indicates a statistically significant difference (p < 0.001) in mean concentration, as determined by Tukey's HSD post hoc test.

Since muscles are the commonly ingestible part of fish, it is crucial for assessing potential health risks from HM. Therefore, this study only focuses on the HM concentration in muscle tissues. The wet weight (ww) concentrations of HMs in the muscles of the sampled fishes along with their comparison with international safety guidelines for fish recommended by several food safety authorities are depicted in Fig. 2. Depending upon food habits, the carnivorous species (*W. attu*) showed a higher tendency to accumulate HMs than the herbivorous species (*C. mrigala*) and omnivorous species (*O. bimaculatus* and *L. calbasu)*. This can be ascribed to the fact that HMs can be biomagnified in the food chain. Therefore, due to their position at the highest levels of the food chain carnivorous species have relatively higher tendency to accumulate HMs [33]. Considering the seasonal variation, HM concentrations were found to be higher in samples during dry seasons due to high evapotranspiration and less flow of water in the river [34,35]. The concentration of Cr was highest in *W. attu* (1.92 mg/kg, ww) during dry season, whereas lowest in *C. mrigala* (0.54 mg/kg, ww) during the wet season (Figure - 2). All fish samples except for *C. mrigala* in wet season accumulate higher or closer (*L*. *calbasu* in wet season) Cr concentrations than the Highest Permissible Limit (HPL) recommended by the food safety authorities (WHO and FAO) [36,37]. Furthermore, the concentration of Cr in the fish obtained from Dhaleshwari River in 2018 was more than 5 times higher, while it was 30–60 times lower than the fish studied from Buriganga River and Turag River in 2021 (Table 2). The Buriganga River was highly polluted because of the discharge of tanning liquor from adjacent tannery industries, for which the concentration of Cr was found to be 30 to 60 times greater than the amount of the present study. Considering these, it can be inferred that the enormous increase in Cr concentration in fish species of the Dhaleshwari River was due to tannery and other industrial pollution.

**Table 2:** Comparison of the amount of the studied metals (mg/kg, ww) obtained in fish species and comparison with amounts from previous literature.

Study Area	Cr	Pb	Cu	As	Cd	References
Dhaleshwari River, Bangladesh	0.54-1.92	0.34-1.42	0.62-1.99	<0.0002	<0.01-0.31	This study
Dhaleshwari River, Bangladesh	0.006-0.159	0.086-0.288	–	<0.005-0.128	0.002-0.019	[38]
Buriganga River, Bangladesh	67.01–187.07	1.20-5.07	2.07-3.51	–	–	[39]
Turag River, Bangladesh	20.18–70.18	4.01–10.18	5.03–10.40	–	–	[39]
Karnaphuli River, Bangladesh	3.36	13.88	12.10	4.89	0.39	[37]
Ganga River Basin	0.25 ± 0.03–1.74 ± 0.31	1.12 ± 0.03–4.77 ± 0.34	0.58 ± 0.09–11.05 ± 2.65	–	0.32 ± 0.07–2.54 ± 0.33	[23]

Pb and Cd were accumulated in higher amounts in *W. attu* (1.42 and 0.31 mg/kg, ww) during the dry season. Remarkably, Cd concentration in analyzed samples was found below the detection limit, BDL (less than 0.1 mg/kg, dw) during the wet season for *C. mrigala*. The LOD of Cd was 0.1 mg/kg; thus, half of the LOD (0.05 mg/kg, dw) was used instead of BDL for risk analysis [40]. While comparing these concentrations in samples with international standards, all the samples contained Pb and Cd that crossed the HPL suggested by FAO. However, according to the HPL proposed by WHO or FAO/WHO, all of them lie within the safe limits (Figure - 2) [34,41,42]. The measured values of Pb and Cd were substantially greater than those reported in the earlier study on Dhaleshwari River, although considerably lower than those found in fish studied from Karnaphuli and Buriganga Rivers (Table 2). The elevated levels of Pb in the Karnaphuli river can be attributed to the operations of the textile, fertilizer, and leather industries in the river's catchment area. This suggests that the contamination of the Dhaleshwari river is similarly caused by industrial runoff. Moreover, the measured Cu concentrations in the analyzed fish muscles varied from 0.62 to 1.99 (mg/kg, ww). The herbivore species (*C. mrigala*) had the lowest concentration during the wet season, while the carnivore (*W. attu*) had the largest concentration during the dry season. All analyzed Cu concentrations lie well within the international limit set by FAO/WHO (Figure - 2) [41,43,44]. Notably, in both seasons, As concentrations were BDL (<0.002 mg/kg, dw) for the sampled fish. A reason for this phenomenon could be attributed to the presence of an insignificant amount or total absence of As in the fish specimens. Hence, 0.001 mg/kg (equivalent to LOD/2) in dry weight was substituted for BDL for subsequent risk evaluation [40].

From a statistical standpoint, there were significant differences in the accumulation patterns of all metals across seasons (p < 0.001, except for As and Cd) and among species (p < 0.05).

### 3.3 Inspection of inter metal relationship

A Pearson's correlation matrix was used to point out the common source of metals in the collected fish species (Table 3). In fish muscles, there are substantial positive correlations in the metals (p < 0.01). Exceptionally, the content of As was not correlated with any of the studied metals, as its concentration remained BDL (′<0.0002 mg/kg, ww) in all samples. Significant positive correlations were observed between Cr and Cu (r = 0.828), Cr and Pb (r = 0.817), and Cr and Cd (r = 0.800) at a significance level of p < 0.01. Conversely, Pb exhibited the highest connection with Pb-Cu (r = 0.799) at a significance level of p < 0.01. Strong correlations among heavy metals (HMs) in fish species indicate that contamination can arise from shared local sources, mutual dependence, and comparable processes of dispersion and/or similar behaviors during their transportation in the research region [45]. Accordingly, it can be stated that Cr, Cu, Pb and Cd might have come from the same sources, such as direct discharge of industrial effluents from Dhaka EPZ, agronomic activities in riverside, municipal waste, etc. in the studied area.

**Table 3:** Correlation Analysis of HMs in sampled fishes from the Dhaleshwari River, Bangladesh.

	Cr	Pb	Cu	As	Cd
Cr	1				
Pb	**.817∗∗**	1			
Cu	.**828∗∗**	0.799**∗∗**	1		
As	B	B	b	b	
Cd	**.800∗∗**	**.753∗∗**	**.753∗∗**	B	1

b. At least one of the factor is constant, so the correlation cannot be found.

∗∗The correlation is statistically significant at the level of 0.01 (two-tailed).

### 3.4 Health risk assessment

Analyzing the HM concentrations in fish species can not solely satisfy its acceptance for safe consumption, as chronic exposure and duration are also important. Hence, it is indispensable to assess potential health risks. The rate of ingestion, along with the duration and exposure, was used to assess the estimated daily intake, noncarcinogenic and carcinogenic risk of consuming the collected fish species.

#### 3.4.1 Estimated daily intake

Humans can be exposed to HMs through consumption, breathing, or the dermal route. Among them, dietary intake is considered the most vital route of exposure [26]. In the light of this, the EDI of the HMs via the consumption of the studied fish samples was assessed and paralleled with the provisional tolerable daily intake (PTDI) recommended by a variety of food safety authorities (Table 4). The computed EDI values were determined to follow a ranking of carnivore > omnivore > herbivore, namely W. attu > O. bimaculatus > L. calbasu > C. mrigala for all the heavy metals, except for Pb. Moreover, values of EDI followed the descending order of Cu > Cr > Pb > Cd > As. While comparing with PTDI values, Pb and Cu had noticeably higher EDI for adults than PTDI whereas Cr, Cd and As had considerably lower EDI than PTDI that indicates potential risk to the population [46,47]. In children, all the EDI for Cr, Pb, Cu and Cd (only for *W. attu)* exceeded the PTDI limit. This can be attributed to their lower bodyweight, which suggests a higher risk due to fish ingestion compared to adults.

**Table 4:** EDI of HMs due to the consumption of fish species and comparison with the PTDI.

Personnel	EDI values (mg/Kg day)					
	HM	W. Attu	O. bimaculatus	L. calbasu	C. mrigala	PTDI∗
Adult	Cr	1.66E-03	1.42E-03	1.31E-03	9.81E-04	3.00E-03
	Pb	1.28E-03	1.05E-03	1.17E-03	7.70E-04	3.57E-04
	Cu	1.77E-03	1.51E-03	1.37E-03	1.12E-03	1.00E-03
	Cd	2.54E-04	1.37E-04	1.39E-04	3.39E-05	8.30E-04
	As	2.26E-07	2.26E-07	2.26E-07	2.26E-07	2.20E-03
Children	Cr	6.65E-03	5.68E-03	5.23E-03	3.92E-03	
	Pb	5.11E-03	4.18E-03	4.67E-03	3.08E-03	
	Cu	7.10E-03	6.06E-03	5.50E-03	4.47E-03	
	Cd	1.02E-03	5.50E-04	5.57E-04	1.36E-04	
	As	9.04E-07	9.04E-07	9.04E-07	9.04E-07	

∗∗ PTDI values set by WHO, USEPA and JECFA [46,47].

#### 3.4.2 Non-carcinogenic risk analysis

This study also implemented the target hazard quotient (THQ) and hazard index (HI), which are globally recognized methodologies for evaluating the non-carcinogenic risk of consumers. The mean concentration of each HM in fish between the two different seasons has been considered to calculate the THQs for consumers considering the long-term exposure. THQ and HI values of the HM through ingestion by an average adult and child in Bangladesh are presented in Table 5. It is important to note that THQ levels do not provide a quantitative assessment of the potential for adverse health effects but rather indicate the amount of risk faced by consumers during prolonged exposure to metals. The threshold value of THQ is 1 and when it crosses unity, it signifies the consumer might pose probable noncarcinogenic risk over the course of the time [26]. In this work, the THQ levels of specific metals acquired by consuming these fishes were found to follow a decreasing order: Cr (0.5541 mg/kg) > Pb (0.219 mg/kg) > Cd (0.254 mg/kg) > Cu (0.044 mg/kg) > As (≤0.001 mg/kg). Upon comparison among fish species, *W. att*u had the greatest THQ for Cr (0.554) and *C. mrig*ala had the lowest THQ for As (≤0.001). The individual THQs of all the studied HMs for an adult were less than unity; thereby, the consumption of these fish species can be considered safe from that perspective. However, child consumers are more vulnerable since the THQ for Cr and Pb (except *C. mrigala*) in all four species exceeded unity. However, considering the combined impacts of all metals, the hazard index (HI) in carnivore (*W. attu*) was higher than threshold 1 (HI = 1.173), which signifies that adult consumers of this species from the studied area might have potential health risks. For the rest of the species, THQ values were below 1. Nevertheless, in the case of child consumers, the severity of risk is quite high, as HI exceeds two to four folds of unity. Moreover, consumers of fish species may be exposed to various toxicants such as PCB, insecticides, fungicides, pesticides, etc., which can further enhance their Hazard Index (HI) [48].

**Table 5:** Calculated THQ, HI, and TR for HMs through consumption of the sampled fish species from the studied area.

THQ							
Heavy metal		Cr	Pb	Cu	Cd	As	HI
Adult	*W. Attu*	0.554	0.319	0.044	0.254	0.001	1.173
	*O. bimaculatus*	0.473	0.261	0.038	0.137	0.001	0.911
	*L. calbasu*	0.436	0.292	0.034	0.139	0.001	0.902
	*C. mrigala*	0.327	0.193	0.028	0.034	0.001	0.582
Children	*W. Attu*	2.217	1.277	0.177	1.017	0.003	4.692
	*O. bimaculatus*	1.893	1.045	0.151	0.550	0.003	3.643
	*L. calbasu*	1.743	1.168	0.137	0.557	0.003	3.605
	*C. mrigala*	1.308	0.770	0.112	0.136	0.003	2.326
TR							
HM		Cr		Pb	Cd		As

Adult	*W. Attu*	8.31E-04		1.09E-05	9.66E-05		3.39E-07
	*O. bimaculatus*	7.10E-04		8.88E-06	5.22E-05		3.39E-07
	*L. calbasu*	6.54E-04		9.93E-06	5.30E-05		3.39E-07
	*C. mrigala*	4.91E-04		6.55E-06	1.29E-05		3.39E-07
Children	W. Attu	3.33E-03		4.34E-05	3.86E-04		1.36E-06
	O. bimaculatus	2.84E-03		3.55E-05	2.09E-04		1.36E-06
	L. calbasu	2.61E-03		3.97E-05	2.12E-04		1.36E-06
	C. mrigala	1.96E-03		2.62E-05	5.15E-05		1.36E-06

#### 3.4.3 Target carcinogenic risk analysis

The target carcinogenic risk (TR) due to Cr, Pb, Cd and As through the consumption of these fish species was estimated (Table 5). Cu is an essential element and not considered as carcinogenic; hence TR for this element was not assessed. USEPA suggested, TR < 10^−6^ as futile; 10^−6^ < TR < 10^−4^ as within a tolerable range and TR > 10^−4^ as unsuitable [49,50]. Values of TR decline from Cr to As for both adult and child consumers (see Table 5). Remarkably, for adults the computed TR of Cr consumer was 8.31E-04, 7.10E-04, 6.54E-04 and 4.91E-04 for the *W. attu, O. bimaculatus, L. calbasu and C. mrigala,* respectively. All these values were several folds higher than the acceptable range of USEPA. Nevertheless, for child consumers, TR for Cr and Cd (Except *C. mrigala*) was significantly higher than adult consumers. Considering food-specific exposure, herbivore consumers might experience less cancer risk than omnivore and carnivore consumers. Apart from that, TR for other HMs (Cd and As) lies between 4.34E-05 to 1.36E-06 and remains within the acceptable range. Based on that, the TR for the consumer of the studied fish species due to metal exposure ought not to be ignored. In addition, considering exposure from other sources such as consuming foodstuffs (i.e., water, rice, vegetables, meat, etc.), dust inhalation or skin contact, which are not considered in this investigation might increase the probable risk of cancer and cause a more complex situation for the consumer.

## 4 Conclusion

The study revealed that fish species sampled were heavily contaminated with toxic HMs (Cr, Pb & Cd). The bioaccumulation of these HMs differed seasonally among species, with carnivorous species exhibiting higher amounts as a result of biomagnification. All fish species examined possessed amounts of Cr, Pb, and Cd that surpassed their respective international safety regulations. The aforementioned metals may have originated from comparable sources, as indicated by the Pearson correlation matrix. Substantial levels of chromium contamination may be attributed to the widespread presence of tanning and other associated industries in the area. Although individual metals may not provide a substantial non-carcinogenic health hazard to adult consumers, the consumption of these metals in combination may pose a health risk, especially for carnivorous fish consumers. Furthermore, the consumption of polluted fish in the examined region may lead to an elevated carcinogenic risk throughout the course of individuals' lifetimes. Unpleasantly, children face a comparatively greater level of non-carcinogenic and cancerous risks from consuming these contaminated substances compared to adults. To ascertain the safety of fish consumption and protect consumers from health risks, it is crucial to quickly establish exact monitoring of the heavy metal contamination in the fish species of the Dhaleshwari River.

## CRediT authorship contribution statement

**Evena Parvin Lipy:** Supervision, Funding acquisition, Formal analysis, Data curation, Conceptualization. **Liton Chandra Mohanta:** Validation, Investigation, Formal analysis, Data curation, Conceptualization. **Dipa Islam:** Writing – review & editing, Supervision, Resources, Methodology, Conceptualization. **Chadni Lyzu:** Validation, Methodology, Formal analysis, Data curation. **Samina Akhter:** Methodology, Investigation, Formal analysis. **Mahmuda Hakim:** Writing – review & editing, Writing – original draft, Validation, Methodology, Investigation, Data curation, Conceptualization.

## Availability of data and materials

The authors confirm that the data supporting the findings of this study are available within the article.

## Ethical approval

The manuscript does not contain any clinical studies. There are no ethical concerns to be reported since the fish were caught by the local fishermen for human consumption. Additionally, during the time of collection, fish samples were not alive. In other words, fish samples were not solely caught for the purpose of the study. Therefore, use of these fish samples in this research does not require any ethical clearance.

## Funding

The authors are cordially expressing their gratitude to the authority of the 10.13039/501100005999Bangladesh Council of Scientific and Industrial Research (BCSIR) for providing necessary funds through the R&D project (ref. no. February 39, 0000.011.14.128.2020/636; December 29, 2020) for this study.

## Declaration of competing interest

The authors declare that they have no known competing financial interests or personal relationships that could have appeared to influence the work reported in this paper.
